# Supplementary material for: Chronic BDNF simultaneously inhibits and unmasks superficial dorsal horn neuronal activity
Source: Sci Rep. 2021 Jan 26;11:2249. doi: 10.1038/s41598-021-81269-6 (PMC7838274; doi:10.1038/s41598-021-81269-6)
Supplement: Supplementary file 1 — Supplementary Information 1. [file 41598_2021_81269_MOESM1_ESM.docx]

**Chronic BDNF simultaneously inhibits and unmasks superficial dorsal horn neuronal activity**

Sascha R.A. Alles^1,2*†^, Max A. Odem^3†^, Van B. Lu^2,4^, Ryan M. Cassidy^5^, Peter A. Smith^2*^

^1^Department of Anesthesiology & Critical Care Medicine, University of New Mexico Health Sciences Center, Albuquerque, NM, USA

^2^Neuroscience and Mental Health Institute & Department of Pharmacology, University of Alberta, Edmonton, AB, Canada

^3^Department of Microbiology and Molecular Genetics, McGovern Medical School at UTHealth, Houston, TX, USA

^4^Wellcome-MRC Institute of Metabolic Science, University of Cambridge, UK

^5^Department of Psychiatry and Behavioral Sciences, Vanderbilt University Medical Center, Nashville, TN, USA

^†^authors contributed equally

**SUPPLEMENTARY MATERIAL**

**Supplemental Figures**

**
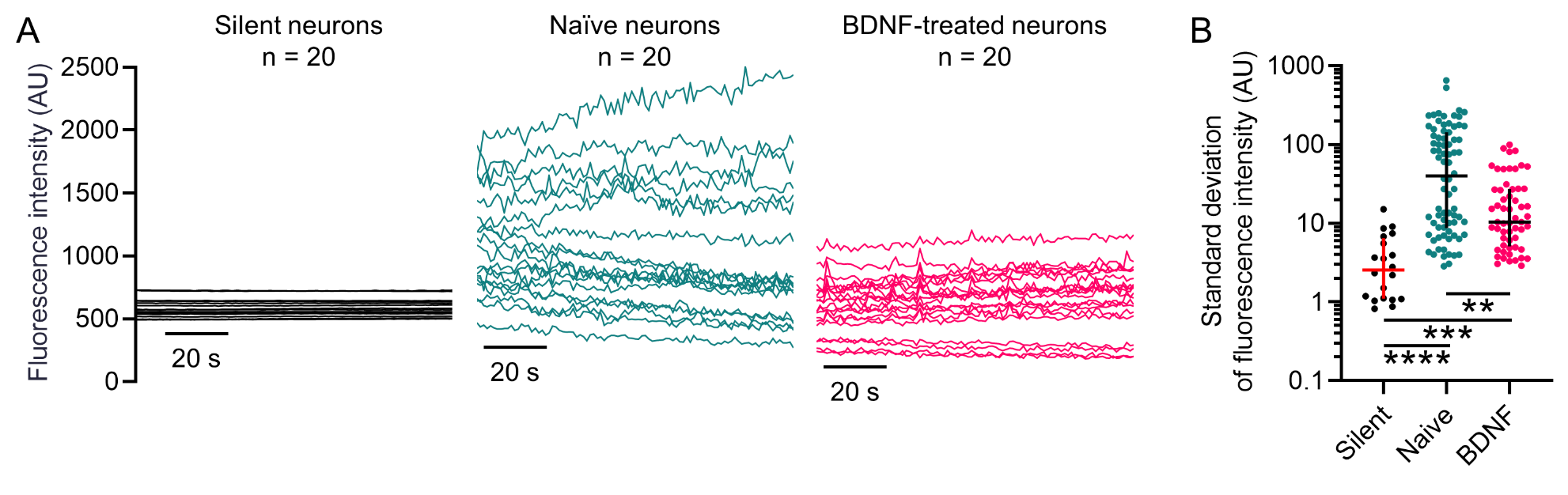
**

**Supplementary Figure 1.** Fluo-4 AM Ca^2+^ imaging in silent, naïve, and BDNF-treated neurons. **(A)** Time-lapse recordings of raw [Ca^2+^]_i_ measured from silent neurons and representative naïve and BDNF-treated neurons. Silent neurons generated smaller fluctuations in [Ca^2+^]_i_ compared to naïve and BDNF-treated neurons. **(B)** Measures of standard deviation also showed that the [Ca^2+^]_i_ signal was less variable in the silent neurons. Error bars represent the median with interquartile range. Comparison made using a Kruskal-Wallis test with Dunn’s post-hoc test. ***P* < 0.01, ****P* < 0.001, *****P* < 0.0001.


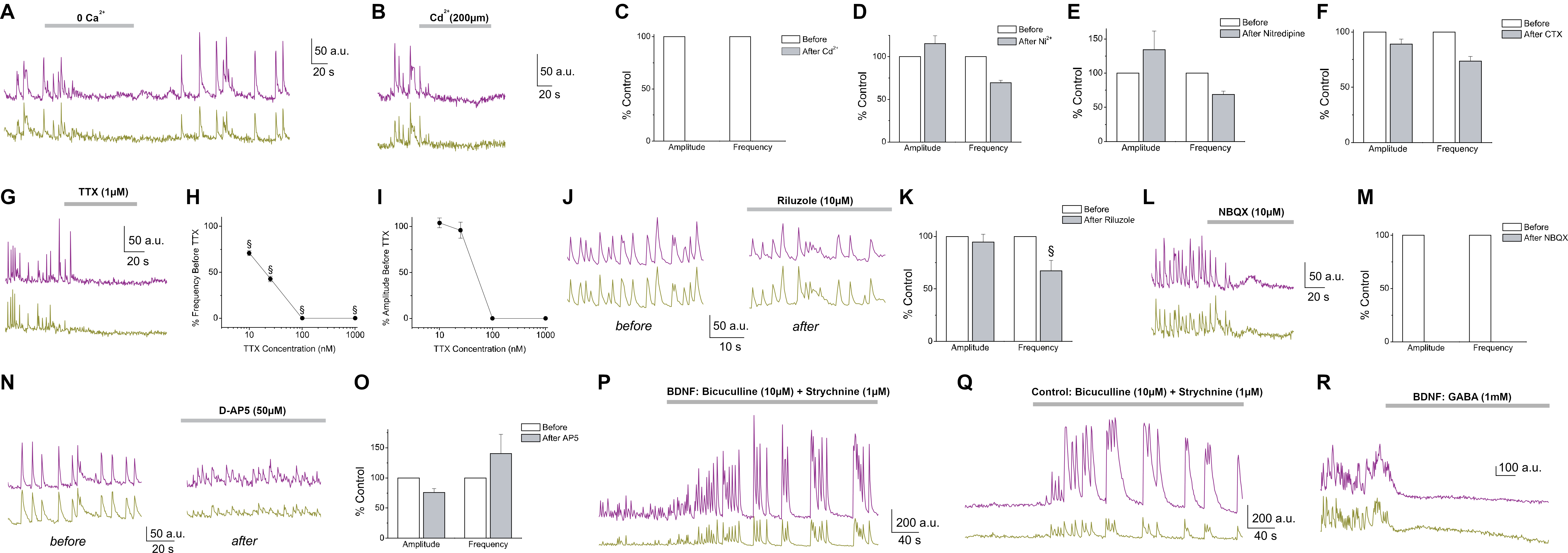


**Supplementary Figure 2.** Pharmacology of BDNF-induced Ca2+ fluctuations. Dependence of BDNF-induced Ca2+ fluctuations on extracellular Ca2+ entry through voltage-gated Ca2+ channels. **(A)** Perfusion of extracellular recording solution free of Ca2+ (0 Ca2+) marked by a thick grey line. Note the complete block of the fluctuations in recorded cells. **(B)** Addition of 200 μM Cd2+, marked by a thick grey line, abolished Ca2+ oscillations in a BDNF-treated slice. **(C)** Measurement of fluctuation amplitude and frequency before and after application of Cd2+. Values normalized to control values obtained before addition of Cd2+. **(D-F)** Effect of 100 μM Ni2+, 1 μM nitrendipine, and 100 nM ω-conotoxin GVIA on BDNF-induced fluctuation amplitude and frequency. Dependence of BDNF-induced Ca2+ fluctuations on TTX-sensitive voltage-gated Na+ current but not persistent Na+ current. **(G)** Addition of 1 μM TTX, marked by a thick grey line, abolished Ca2+ fluctuations in a BDNF-treated slice. **(H)** Concentration-inhibition curve for increasing concentrations of TTX on Ca2+ fluctuation frequency. **(I)** Concentration-inhibition curve for increasing concentrations of TTX on Ca2+ fluctuation amplitude. Error bars indicate standard error of the mean. For paired t-test, § = p<0.001. **(J)** Sample synchronous Ca2+ fluctuation traces from a BDNF-treated slice before (left) and after (right) application of 10 μM riluzole. **(K)** Effect of riluzole on average Ca2+ fluctuation amplitude and frequency. BDNF-induced Ca2+ fluctuations mediated by AMPA/kainate glutamate receptors. **(L)** Addition of 10 μM NBQX, marked by a thick grey line, abolished Ca2+ fluctuations in a BDNF-treated slice. **(M)** Measurement of fluctuation amplitude and frequency before and after application of NBQX. **(N)** Sample fluorescent Ca2+ traces from a BDNF-treated slice before (left) and after (right) application of 50 μM AP5. **(O)** Effect of AP5 on average Ca2+ fluctuation amplitude and frequency. Amplification of BDNF-induced Ca2+ oscillations by pharmacological removal of inhibition and suppression of oscillatory activity by GABA. **(P)** Addition of 10 μM bicuculline and 1 μM strychnine to a BDNF-treated slice produced robust fluctuations larger in amplitude but slower in frequency than the spontaneous Ca2+ fluctuations observed before antagonist application. **(Q)** Addition of 10 μM bicuculline and 1 μM strychnine to a control DMOTC slice produced similar robust fluctuations as those observed in P. **(R)** Application of 1 mM GABA, marked by a thick grey line, stopped BDNF-induced Ca2+ fluctuations. Average values represented. Error bars indicate standard error of the mean. For paired t-test, § = p<0.001, n=5-20 cells per condition.


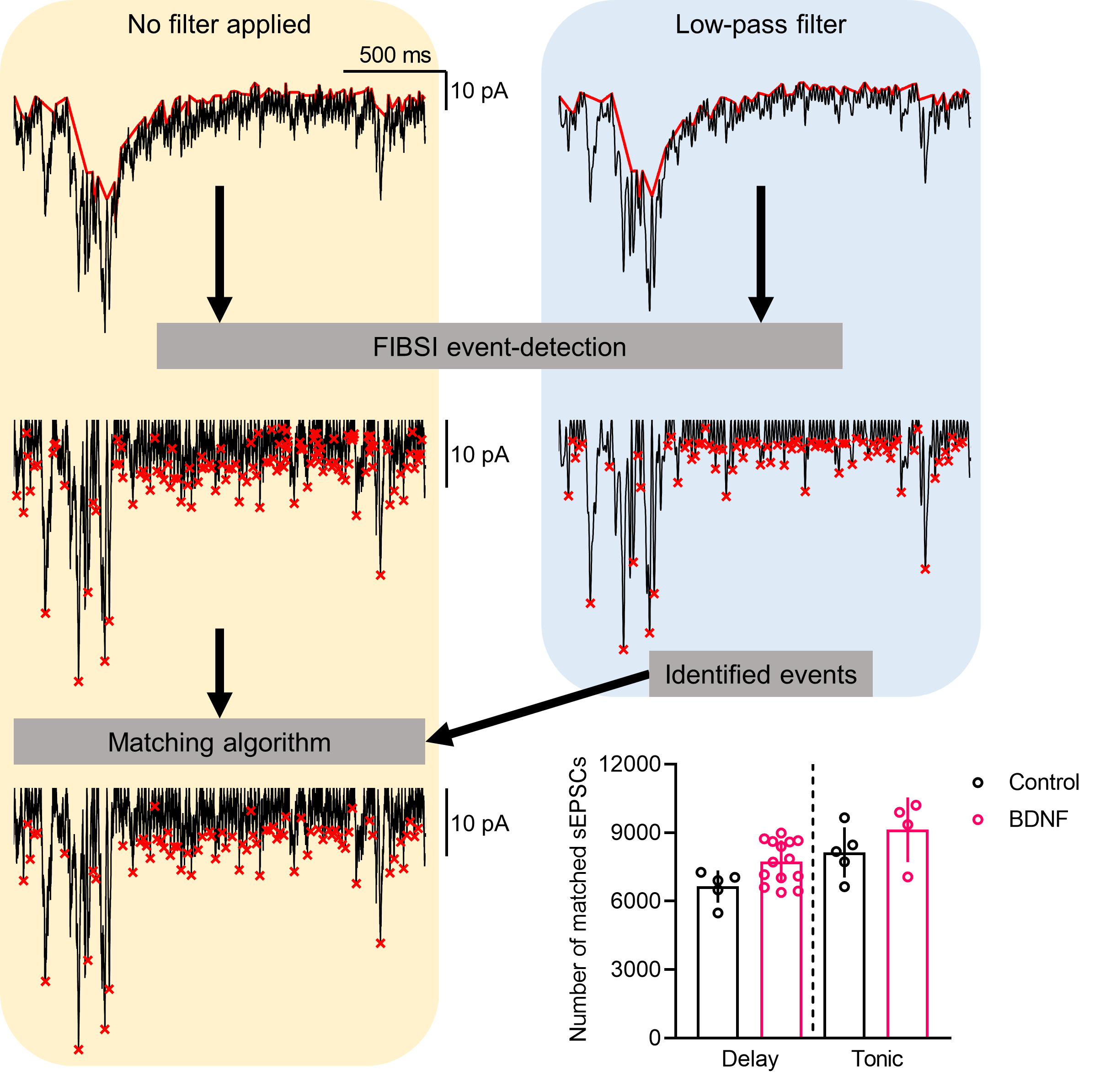


**Supplementary Figure 3.** Matching filtered spontaneous EPSCs detected by FIBSI with the raw recordings. All raw recordings were first analyzed by FIBSI without pre-processing filtering. All recordings were analyzed again following application of a low-pass filter with α = 0.05. The output text files generated by FIBSI containing descriptive parameters (e.g., start time, peak time, amplitude, etc.) for each detected event were used as input to a custom Python script. The matching algorithm matched events detected in the filtered recordings with their corresponding events in the unfiltered recordings. Events were matched based on peak event time and amplitude. Unmatched events were discarded from further analyses. The total number of matched sEPSCs in the delay and tonic neurons are shown.


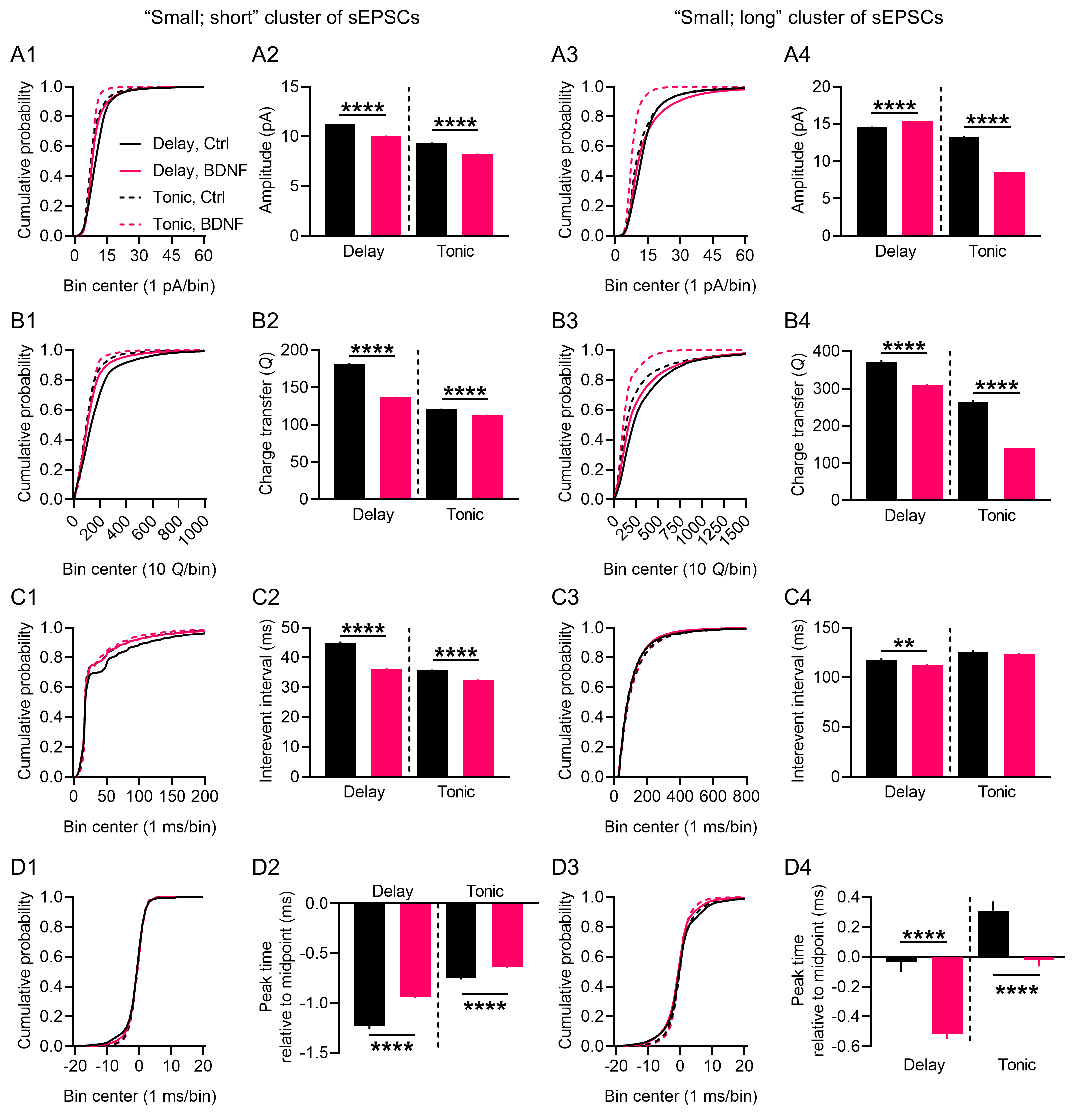


**Supplementary Figure 4.** Effects of BDNF on the two small clusters of spontaneous EPSCs in delay and tonic-firing superficial dorsal horn neurons. Consistent, statistically significant effects of BDNF were observed for the ‘small; short” cluster of sEPSCs in the delay and tonic neurons; amplitudes were smaller **(A1-A2)**, charge transfer values were smaller **(B1-B2)**, within cluster interevent intervals were shorter **(C1-C2)**, and kinetics were slower **(D1-D2)**. Nuanced effects of BDNF were observed for the “small; long” cluster of sEPSCs in the two neuron types; sEPSC amplitudes were increased in delay neurons and decreased in tonic neurons **(A3-A4)** while charge transfer values were decreased in both types of neurons **(B3-B4)**. Interevent intervals in the delay neurons were significantly decreased, but not in the tonic neurons **(C3-C4)**. The sEPSC kinetics were significantly faster in the BDNF-treated delay and tonic neurons **(D3-D4)**. Small; short sEPSC cluster sample sizes: delay neurons control n = 20062, BDNF n = 69670; tonic neurons control n = 25235, BDNF n = 22086. Small; long sEPSC cluster sample sizes: delay neurons control n = 7638, BDNF n = 22391; tonic neurons control n = 7160, BDNF n = 5851. Comparisons between means in the control and BDNF conditions were made using Brown-Forsythe and Welch’s ANOVA tests and Games-Howell post-hoc test. ***P* < 0.01, *****P* < 0.0001.

**Supplementary Video 1.** Representative Fluo-4 Ca^2+^ confocal imaging videos of **(A)** naïve and **(B).** BDNF-treated spinal organotypic slice. Each organotypic slice was incubated for 1 h prior to imaging with the fluorescent Ca2+-indicator dye Fluo-4-AM (Molecular Probes, Invitrogen, Carlsbad, CA, USA). The conditions for incubating the dye were standardized across different slices to avoid uneven dye loading. Full frame images (512 x 512 pixels) in a fixed xy plane were acquired at a scanning time of 0.8-1.08 s/frame (48). In some experiments, images were cropped to accommodate faster scan rates. Selected regions of interest were drawn around distinct cell bodies depicting neurons based on morphology as described previously (49).

1. <insert “Supplementary Video 1A-Naïve.avi”>
2. <insert “Supplementary Video 1B-BDNF.avi”>
